# The Patriarchy Index for Asia: A new tool for subnational analysis of gender inequalities

**DOI:** 10.1371/journal.pone.0339587

**Published:** 2026-01-06

**Authors:** Mikołaj Szołtysek, Bartosz Ogórek, Mateusz Grzyb, Siegfried Gruber

**Affiliations:** 1 Institute of Sociological Sciences, Faculty of Social and Economic Sciences, The Cardinal Wyszynski University in Warsaw, Warsaw, Poland; 2 Institute of History, Polish Academy of Sciences, Warsaw, Poland; 3 Southeast European History and Anthropology, Institute of History, University of Graz, Graz, Austria; Utah State University, UNITED STATES OF AMERICA

## Abstract

Gender inequality remains a persistent barrier to development in many parts of Asia, yet the domestic foundations of this inequality—particularly within family systems—are often neglected in global measurement tools. This study introduces the Patriarchy Index (PI), a new metric constructed from census microdata to capture gendered power hierarchies in families across 22 Asian (including Egypt, treated here as part of the classical Asian patriarchal belt) countries and 652 subnational administrative units. Of particular interest to development practitioners and scholars, the PI offers a scalable, low-cost tool for subnational diagnostics, especially where standard measures of gender equality are missing or fail to reflect private-sphere constraints on women’s autonomy. Our study addresses whether domestic arrangements—such as patterns of co-residence, marriage timing, and age-based authority—can reliably capture institutionalized patriarchy, and what regional variation these patterns reveal. Using harmonized IPUMS-International census microdata and eleven theoretically grounded indicators, we construct a multidimensional composite index empirically validated through convergence with existing gender measures, divergence from unrelated metrics, and correlation with gendered development outcomes. Our study finds that family-based patriarchy is spatially clustered and highly variable at the subnational level. It further shows that higher PI values are significantly associated with reduced relative female labor force participation, even after controlling for structural variables such as gross national income and urbanization. These results underscore the PI’s value as a complementary measure: it captures dimensions of gender inequality that remain invisible to public-facing or outcome-based indicators and helps bridge the gap between domestic constraints and broader patterns of disenfranchisement. In contexts where legal reform and female empowerment are pursued without addressing household-level structures, the PI offers a diagnostic that speaks directly to the architecture of family systems—illuminating where and how deeper constraints on gender equality endure.

## Introduction

Patriarchy, a system in which men have greater access to power and resources than women, and some men dominate others [1–3], has historically existed in various forms in Europe and Asia [2,4–9]. Although some form of patriarchy likely exists worldwide, the Asian region is often portrayed as having deeply entrenched patriarchal beliefs that have persisted for centuries and have not fully dissipated even with modernisation and industrialisation [10–15; also [16]]. From a comparative global sociology perspective, many Asian countries, especially in the Middle East, have had and continue to exhibit some of the highest levels of patriarchy in the world [e.g., 3,17–20]. In these regions, the rudimentary patriarchal system continues to dominate, influencing or underpinning every system, structure, and relationship. It is perpetuated through familial arrangements, modes of production, state-regulated laws and policies, control of sexuality, and monopolies in religion and culture, all of which limit the autonomy and agency of women living under such social conditions.

While Asia is not unique in experiencing a crumbling patriarchal order, the extent of this change, its driving forces, and its impact remain contested by scholars and policymakers. Although the “long patriarchal night of humanity” may indeed be dawning over the world [2, p.130], in the Asian part of the Global South this progress may still be visible only to a minority due to entrenched patriarchal structures [21]. From a social-policy standpoint, understanding and monitoring these broader social structures of patriarchy is essential for the international community to improve gender equality and empowerment across the broad Asian hemisphere, thus meeting the Millennium Development Goals and the Gender 2030 Agenda [e.g., 18,22–25].

However, despite recognizing the pervasive role of patriarchal structures as barriers to a more equitable and just world in Asian societies, little effort has been made to develop multidimensional measurement tools that capture this social reality *glocally*—both on a large comparative scale and at a fine-grained level. Although literature on women’s empowerment and economic positions in Asia has long underscored patriarchy through concepts such as Caldwell’s *patriarchal belt* [26], Moghadam’s *patriarchal gender contract* [27], and Kandiyoti’s *classic patriarchy* [3], these have seldom been quantitatively operationalized or comprehensively measured [18,23,28–30; cf. 31].

This paper addresses the gap by contextualising and quantifying family patriarchy, a social system underlying many gender-inequitable practices in Asian societies (although our title refers to Asia, the dataset includes one transcontinental case—Egypt—whose inclusion is justified by its historical and demographic alignment with the classical patriarchal belt of the Middle East; for the sake of consistency, we use ‘Asia’ as a collective label for the entire sample). In doing so we consider family patriarchy a crucial indicator that is equally, if not more, important than most measures of women’s empowerment and gender inequality previously used in economy and politics. This is because, as we elaborate in due course, it pertains to family norms, the relationships between husband and wife, gender roles and authority models, all of which are believed to be one of the values most resilient to change in societies exposed to modernizing institutions [25,32,33]. By suggesting this extension in current diagnostic tools, this paper capitalizes on the recent global revolution in the availability of harmonised census microdata. These data enable the operationalisation of the familial and demographic characteristics of patriarchy, providing input variables for developing a tool to measure and monitor the gendered distribution of power and hierarchy in the domestic/family sphere in Asia at low cost but with high efficiency.

Our paper makes three key contributions to the existing literature. First, building on the work of Gruber and Szołtysek [6–7], we introduce and assess a new measure of gender inequality — the Patriarchy Index (henceforth, PI) — applied here to the Asian context. PI incorporates dimensions and variables not captured by previous indices and is the only indexed measure that explicitly targets social practices in family, household, and demographic domains that shape women’s empowerment and gender inequality [28,29,34,35]. The index is theoretically applicable to any society where basic microdata are available and can be readily calculated from census or census-like datasets since the 1970s. As we demonstrate, it is particularly suited to Asian settings, where both gendered institutions — norms, traditions, and values — and locally rooted family practices remain especially influential.

The second major contribution of this study is to demonstrate the Index’s capacity to extend research on Asian gender regimes both geographically and conceptually. By applying PI to harmonized IPUMS-International census microdata (including one household survey), we capture subnational variation in gender inequality with a level of consistency and detail not previously achieved. Never before has family patriarchy — and indeed, any other measure of gender (in)equality — been reported and analyzed at such scale and granularity across the region. Not only is the PI represents a rare empirical tool for identifying gender discrimination below the nation-state level, but also – as we demonstrate – it yields distinctive patterns not captured by existing indices. As such, it provides a replicable framework for localized, cross-national comparison, with the potential to challenge essentialist assumptions that portray Asian gender regimes as uniform or culturally monolithic, by illuminating the internal variation in patriarchal structures across the region [23,26,36,37].

Third, we recognize that the public–private divide [38] is culturally constructed and varies across time and context. Changes in women’s demographic behaviors — such as residence patterns — can shape their participation in education and labor markets, while gender norms often shift across domains in tandem [33,39]. In this light, PI not only extends empirical reach beyond existing measures, but also has important implications for gender stratification theory by revealing the context-sensitive synchrony between familial and extra-familial inequalities [17,40,41].

## Rationale and significance

According to the large body of literature, in many areas of the Global South, traditional patriarchal practices in grassroots institutions like families and households continue to exacerbate gender bias and discrimination, significantly impacting long-term developmental outcomes for individuals and populations [15,22,42–46]. This is particularly true for societies stretching from North Africa, through Western Asia and the Middle East, to Central and South Asia. In these regions, patriarchal cultures shape experiences and interpretations of gender, hierarchy, and power through binary thinking and hierarchical structures, assigning unequal gender and age-graded roles and maintaining power dynamics between hierarchically structured groups such as men and women, or adults and children [3,9,22,47–49]. This value system not only defines possible roles and influences life course trajectories but also impacts women’s well-being and life choices. This is evident through the continued presence of patriarchal kinship and household organization, high fertility and early marriage, high maternal and infant mortality rates, female illiteracy, lower educational enrollment, reduced labor force participation, and the lack of political participation and rights for women [2,9,15,18,22,23,50,51].

Patriarchal discourses and practices are typically acquired during early upbringing within the family and through significant others from close kin [52–55]*.* Existing research has shown that early life exposure to patriarchal norms within the family is a risk factor for experiencing various forms of violence (emotional and physical) in intimate relationships during adolescence. Additionally, it can lead to the development of reinforced traditional binary gender roles and stereotypes in later life [55–57].

Meanwhile, most assessments of women’s status, autonomy, and gender inequality in Asia focus on outcome measures and outputs such as female participation in the public sector, literacy rates, education levels, labor force participation, health, and income [58–61]. Over the past two decades, efforts have been made to implement additional specific metrics to examine institutional barriers perpetuating gender inequality by considering women’s standing relative to men in social norms, traditions, and family law [62–64]. These efforts typically use data from national or international agencies, usually at the country level, with no aim of capturing family patriarchy [65–67].

Although enduring traditions, customs, and formal or informal laws strongly shape gender-inequitable practices, they are neither the sole nor the most immediate sources of gendered and age-based hierarchies. These hierarchies are also reproduced in everyday social microstructures, where domestic groups serve as key sites for transmitting and reinforcing norms and values. Yet existing measures such as the Social Institutions and Gender Index (SIGI), while presented as indicators of “long-lasting social institutions defined as societal practices and legal norms that frame gender roles” [64, p.29], largely capture only the legality of practices or the norms surrounding them, rather than their actual prevalence. This gap underscores the importance of examining families directly: as the primary social unit, they shape beliefs and behaviors, perpetuate values related to sex- and age-based hierarchies, power, and freedom, and convey gendered discourses that either reinforce or challenge patriarchal culture [33,34,52,54,55,68,69].

Most existing global measures alluded to so far, typically operate at the country level, despite high levels of regional inequality within most countries. For example, the otherwise powerful SIGI accounts for institutions only at the national level. While this makes sense for state-wide institutional contexts, it is clear that people do not uniformly follow the legal-normative order everywhere [70], and the strength of these *institutions* is often not uniform across all regions within countries. The lack of small-area indicators is a significant barrier to developing and evaluating gender equality programs and policies.

Finally, most existing metrics hastily presume a historical shift from private, family-centered patriarchy to public, state-centered patriarchy when discussing inequality structures [71–73]. As a result, the existence of gender inequality in more hidden domains is not accounted for, leaving aside the actual underpinnings (inputs) of these inequalities. Meanwhile, the problem of women’s autonomy in truly patriarchal societies outside the Global North, particularly in Asia, cannot be reduced to the male-oriented concept of status based on human capital outcomes or output [45,74,75]. Many analysts point to the role of family and household as crucial sites for ongoing empowerment [42,46,75–82,83], arguing that neglecting the subtleties of women’s autonomy in the household obstructs a full understanding of gender inequality. In fact, extant research provides substantial evidence that domestic inequalities persist even in countries with high levels of female participation in the public sector and other areas of empowerment [84–89].

In light of these points, a comprehensive monitoring of women’s autonomy, status, and experiences of inequality requires indicators that address gendered and generational power distribution and hierarchy also in the domestic sphere, and at the same time allowing researchers to move below national aggregates. This is particularly urgent for societies where families continue to be major socializing entities and where gender inequality issues are deeply rooted and reinforced through cultural scripts and familial traditions [15].

## Materials and methods

### Data

**Fig 1:**
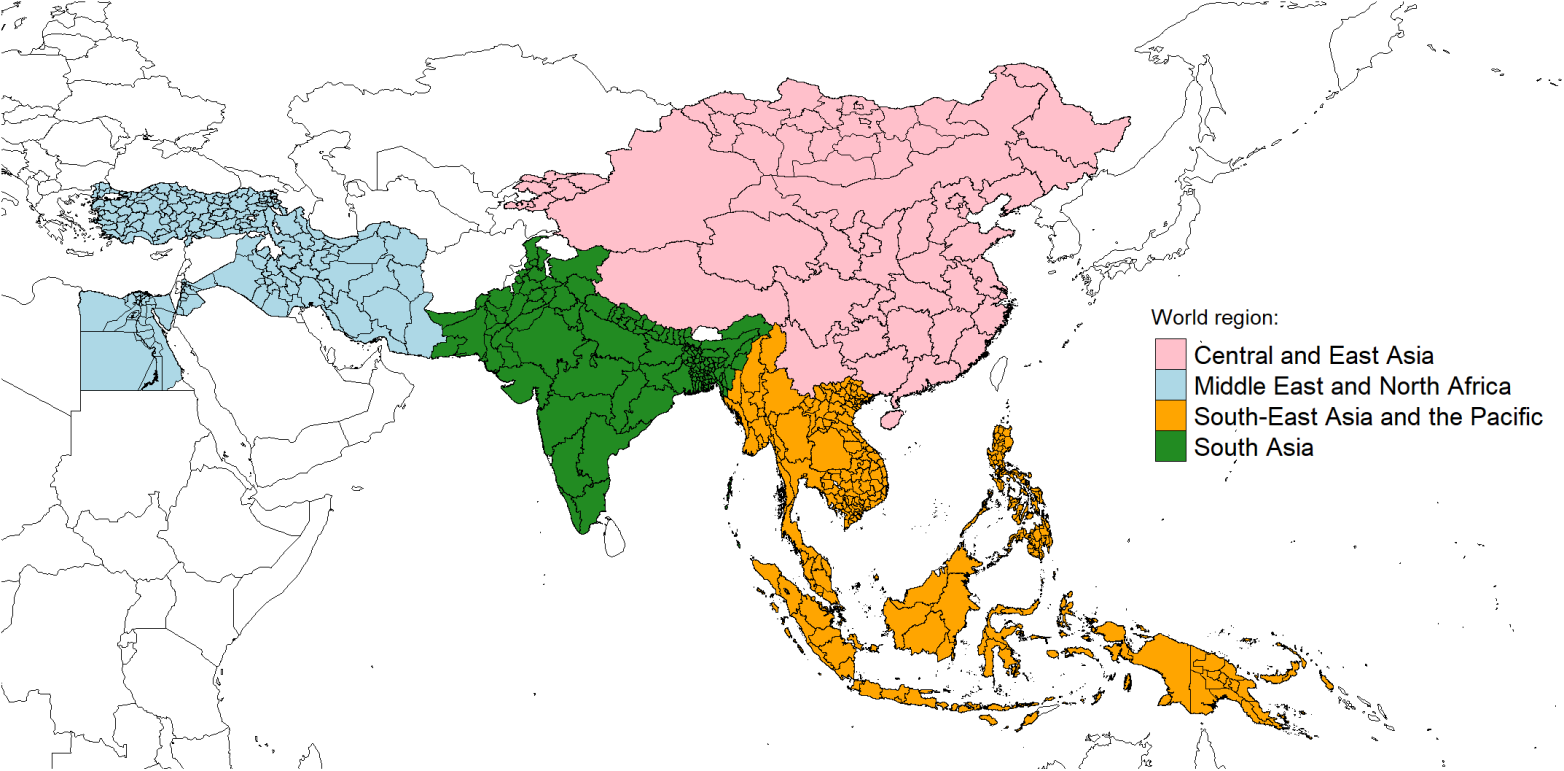
Subnational data distribution by macro-regions. Notes: Data source and regional divisions as presented in Table 1. Country and subnational boundaries were derived from public domain data provided by Natural Earth (https://www.naturalearthdata.com) via the rnaturalearth R package [59]. Data are in the public domain and therefore compatible with the CC BY 4.0 license.

This study uses harmonised representative census microdata samples from 22 Asian countries (including Egypt, for reasons outlined earlier) provided by IPUMS-International (including one survey data). The dataset includes countries traditionally associated with the *classical patriarchal belt* (i.e., Bangladesh, China, Egypt, India, Iran, Iraq, Jordan, Pakistan, and Nepal), as well as nations occasionally categorised as belonging to this group (i.e., Armenia, Indonesia, Malaysia, Vietnam, Mongolia, and the Kyrgyz Republic) [3,15,18,23,26,90–95]. It also incorporates all other Asian countries available from IPUMS-I, predominantly from Southeast Asia. The census and survey data span from 1997 to 2015 and cover nearly 106,406,041 individuals across 652 subnational administrative units (Table 1 and Fig 1). When multiple samples were available, the latest census (or survey, as in the case of India) was selected, provided it met specific structural standards. Additionally, datasets were chosen to minimize the time span between years of enumeration, ensuring the data could be used as pooled-time cross-sections, with an average absolute difference of five years. For this reason, although more recent censuses were available for three countries—Cambodia and Vietnam (2019, 11 and 10 years respectively after our chosen reference point), as well as Mongolia (2020, twenty years after our chosen reference point)— we deliberately prioritized coherence over recency and refrained from including them, in order to preserve greater comparative consistency across the dataset as a whole. The division into subnational units was based on those available in IPUMS-I, selecting divisions that offered the best compromise between detailed spatial distributions, comparability of the number of divisions between countries, and obtaining small area samples of sufficient size to derive specific indicators. Although census years vary slightly across countries, the harmonisation standards applied by IPUMS-I ensure full comparability of variables, so these minor temporal differences do not affect the construction or validity of the Patriarchy Index.

**Table 1:** Characteristics of the samples.

Country	Enumeration year	Sample fraction (%)	Sample size	No. of regions	Mean sample size (level 1 or 2)	IPUMS GIS levels	Type of region
Armenia	2011	10	301831	11	27439	GEO1_AM2011	provinces
Bangladesh	2011	5	7205720	64	112589	GEO2_BD2011	districts
Cambodia	2008	10	1340121	24	55838	GEO1_KH2008	provinces
China	2000	1	11804344	31	34719	GEO1_CN2000	provinces
Egypt	2006	10	7282434	27	269720	GEO1_EG2006	governorate
India	2009	0.04	560741	31	16021	GEO1_IN2009	state
Indonesia	2010	10	22928795	33	694812	GEO1_ID2010	provinces
Iran	2011	2	1481586	31	47793	GEO1_IR2011	provinces
Iraq	1997	10	1944278	15	129619	GEO1_IQ1997	governorate
Jordan	2004	10	510646	12	42554	GEO1_JO2004	governorate
Kyrgyz Republic	2009	10	564986	9	62776	GEO1_KG2009	region
Laos	2015	10	627489	18	34861	GEO1_LA2015	provinces
Malaysia	2000	2	435300	15	29020	GEO1_MY2000	state
Mongolia	2000	10	243725	21	11606	GEO1_MN2000	provinces
Myanmar	2014	10	5032818	15	335521	GEO1_MM2014	state
Nepal	2011	12	3238842	14	231346	GEO1_NP2011	administrative zone
Pakistan	1998	10	13102024	27	485260	GEO2_PK1998	provinces
Papua New Guinea	2000	10	520609	20	26030	GEO1_PG2000	provinces
Philippines	2010	10	9411256	85	110721	GEO1_PH2010	provinces
Thailand	2000	1	604519	5	120904	GEGNTH	provinces
Turkey	2000	5	3388218	81	41830	GEO1_TR2000	provinces
Vietnam	2009	15	14177590	63	225041	GEO1_VN2009	provinces

Notes:

Data source, see [95]. See also S1 Data.

Spatially harmonized first-level geography (GEOLEV1) from IPUMS-I was used. Exceptions: Bangladesh (LEV2) uses 64 districts instead of 7 (1M persons per region). Pakistan (LEV2) shifts from 5 large regions (2.6M persons per region) to 27 administrative units. Thailand consolidates 76 provinces (8K persons per region) into 5 broader regions for comparability.

However, certain countries of the *classical patriarchal belt* are not included, either due to data availability (Algeria, Tunisia, Libya, Saudi Arabia, Syria, Lebanon, Yemen, and Afghanistan are not in IPUMS-I) or incompatible data structures for calculating the focal measure (Israel, Palestine, and Morocco; data from the Demographic Health Surveys are not considered in this paper for the same reasons).

All IPUMS data uniformly categorise individuals into “coresident domestic groups” (households), and capture key variables such as the relationship to head of household, age, gender, and marital status, as well as variables that identify family-kin relationships within household groups [96]. These variables enable standardised demographic analyses in different settings, and thus allow for a comparative study of family structures at the meso-level, including our preferred metric of family patriarchy. All regional data are geo-referenced.

### Samples’ characteristics

While patriarchal systems are not unique to Asia [97–99], our focus reflects both conceptual and methodological considerations. In contrast to Sub-Saharan Africa — where family systems differ substantially [100,101] and available large-scale data are primarily derived from Demographic and Health Surveys that do not fulfil all the microdata requirements for computing PI — Asia offers greater compatibility with census-based datasets (e.g., IPUMS) and a historically distinct configuration of kinship and domestic organization.

All studied populations have a recent history as predominantly kinship-based rural societies, with varying degrees of patrilineal (descent traced through the male line) and patrilocal (marriage residence with the husband’s family) practices, alongside bilateral kinship (descent recognised through both parents) in some areas [102]. The societies considered here display diverse family structures, including nuclear, stem, and joint families, often with marked regional heterogeneity [103]. Hierarchical, non-egalitarian family systems have long been present and often continue to exist across many areas of the sample, with India and China epitomizing the strongest patriarchal systems [10,20]. The *classic patriarchy belt* societies in the Asian sample, characterized by extended family systems and persistent clan structures [15,25,90], contrast with Southeast Asia’s more egalitarian societies [104].

All IPUMS populations considered here have completed the first demographic transition [105,106]. Since the 1960s (or earlier in some cases), they have experienced declines in teenage marriage, rising ages at first marriage, and overall reductions in marriage rates [107–110]. These trends coincide with increasing life expectancy and a general decline in fertility [50,111,112]. At the time of the census, however, only five of the 22 nations had fertility below replacement level (including China’s critically low rate), underscoring that while fertility decline is widespread, entry into the second demographic transition remains limited. A fuller convergence of these societies with Western cultural and behavioral models of the second demographic transition is therefore contested in the scholarship [105,113,114].

As fertility has declined in Asia, households have become smaller but not necessarily simpler. The probability of living with non-primary relatives (i.e., in multigenerational households) has not changed significantly or has even increased around the census dates in most countries analyzed here, including China (own calculations based on [115]; see also [116–119]. Although ongoing changes may weaken family ties in Asia, intergenerational relations remain relatively strong in this part of the.

While various policies, legislative reforms, and advocacy efforts have influenced family dynamics in the focal areas [2,11,17,23,44,50,78,90,114,119–121], these initiatives have had complex effects—sometimes undermining patriarchal family structures and sometimes supporting modernized forms of patriarchy, but otherwise maintaining the family’s regulatory role and influence in gender relations and social reproduction [11,85,90,122–124].

The sample exhibits diverse religious affiliations across countries and regions. This includes three major categories of Hinduism, Islam and Buddhism, as well as Christianity (both Eastern and Western) and various forms of traditional/folk spiritual practices. China is predominantly considered Buddhist, except for the Muslim-majority region of Xinjiang, due to Buddhism’s historical and cultural influence. In Vietnam, traditional/folk spiritual activities are predominant and reported as *folk religion*. All these religions can directly or indirectly influence the level of patriarchy in a society, although the influence of religious cultures may be neither clear nor uniform [125–127].

Last but not least, although our data exclude economic powers like Japan, South Korea, and Hong Kong, there are strong economic disparities among the Asian countries and regions considered. The socioeconomic context of patriarchy ranges from the more developed economies of Iran, Malaysia, Turkey, and Thailand to the low economic productivity settings of Nepal and Papua New Guinea, with various intermediate levels. These disparities reflect diverse factors, including natural resources, industrialization levels, political stability, and economic policies around the respective census dates.

### Measurement properties of the Patriarchy Index

#### Construction.

To quantify familial patriarchy across the Asian dataset, we rely on the Patriarchy Index (PI) developed by Gruber and Szołtysek [6–7]. Drawing on ethnographic and historical-anthropological accounts of hierarchical kinship systems in Eastern Europe, the Balkans, and the Middle East, the authors formulated a set of items for cross-cultural comparison that reflect the multifaceted manifestations of patriarchal order. Based on their comprehensive screening of the literature, they conceptualized patriarchy as a historically persistent and institutionally embedded system of male dominance, operating not only through the subordination of women, but also through age-based hierarchies among men [1,2,9,49,128–130]. A critical insight shared by these accounts—as well as by extensive literature on kinship and gender relations in various parts of Asia—is the recognition that patrilineality, patrilocality, endogamy, and age- and sex-stratified authority function as core structural logics through which patriarchy is reproduced within family organization [3,28,99,104,131,132].

To render these dimensions measurable globally and at high resolution, Gruber and Szołtysek systematically mapped them onto observable indicators by operationalizing the original theoretical constructs in alignment with the informational structure of census microdata—a uniquely suited source for capturing large-scale, standardized information on family structures across time and space [96,133]. As such data typically contain no direct measures of power, norms, or ideology—but instead offer granular detail on household composition, kin co-residence, marital timing, and age-sex distribution of statuses—the authors identified patterned regularities through which patriarchal logics become empirically traceable.

**Fig 2:**
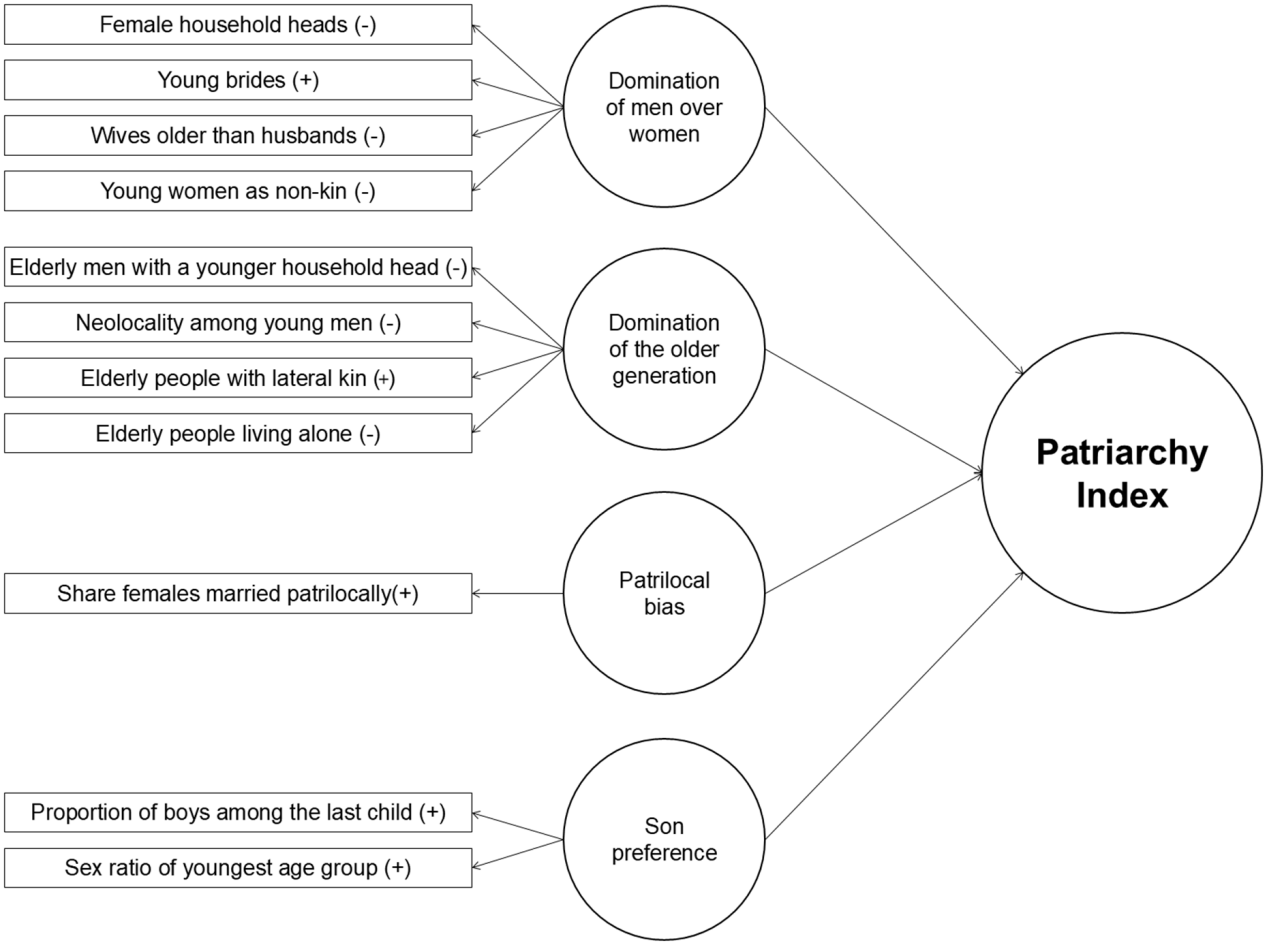
The four major dimensions of patriarchy and their input variables. Notes: Indicators (from top to bottom): (1) female household heads aged 20+ among all adult heads, (2) ever-married women aged 15–19, (3) couples in which the wife is older than the husband, (4) women aged 20–24 living only with non-kin, (5) men aged 65+ in households headed by a younger male relative, (6) male heads aged 20–29 living only with their immediate family, (7) persons aged 65 + living with at least one lateral kin, (8) persons aged 65 + living without relatives or a spouse, (9) ever-married women aged 15–30 living with an adult male kin of the husband or his mother, (10) boys among children aged 10–14, and (11) child sex ratio (boys to girls) aged 0–4. For details on the measurement of these components and IPUMS-based adjustments, see Table A in S1 Text. (+/−) indicates the expected direction of each component’s relationship with societal patriarchy levels. Arrows denote the suggested relationships between constructs in the model: reflective lower-order constructs and the formative higher-order construct.

In order to distil theory into census-derived signatures, Gruber and Szołtysek formulated **eleven imperatives** of patriarchal family organization—structural indicators designed to capture latent dimensions of gendered and generational inequality within domestic units. These principles include: 1) women do not head households; 2) they marry early, often at or soon after puberty; 3) they are always younger than their grooms; 4) they stay with their natal families until marriage; 5) young men do not become household heads while seniors are alive; 6) sons of living fathers do not establish independent households; 7) some men remain in the family household permanently; 8) elders are not left to care for themselves; 9) at marriage, girls move into their husband’s or father-in-law’s household; 10) parents prefer to raise sons over daughters; 11) girls receive less care than boys (Fig 2). These imperatives reflect aspects of patriarchy that large-scale census microdata can capture, even within their limited scope. Where these key features coalesce, they form a tightly integrated structure of gendered and generational inequality—not just scattered traits, but a coherent *ideal type* of familial patriarchy, structurally embedded and demographically visible.

Accordingly, the eleven indicators were defined as input variables to capture specific aspects of patriarchal structures (Fig 2), with the original computation of PI [6] adjusted for minor information-structure differences in IPUMS-I (see S1 Text), entailing little or no information loss. For each input variable a 10-point scale was used, measuring each population (national/regional/local) on a 0–10 performance scale. The first nine indicators are benchmarked against the highest possible outcome, with the best region scoring 10 and others scoring lower (0 for a natural lowest value). The last two variables, due to their distinct ranges, use neutral proportions of 0.51 and 105 as minimum values, with a similar approach for maximum values.

According to the reflective-formative Type II model [134], the eleven input variables serve as reflective indicators organized hierarchically into four domains or sub-indices: *domination of men over women*, *domination of the older generation*, *patrilocal bias*, and *son preference*. Collectively, these sub-indices form the higher-order construct of familial patriarchy, encapsulating its influence on potential outcome variables [135,136].

The overall patriarchy measure, as a higher-order construct, is a nested average of eleven indicators across four domains, ranging from 0 to 40 patriarchy points. Since each domain uniquely contributes to the construct and is non-interchangeable, the final PI is computed by giving equal weight to the four dimensions and the indicators within each dimension [137]. While the component indicators may vary in their representation of patriarchy, the values of the domains (sub-indices) have been scaled and normalized to ensure unidirectionality. Additionally, when computing age- and sex-specific indicators, standardization by the average age-sex structure of all national populations included in this research was used to account for demographic differences.

PI, theoretically applicable to any human society, is distinguished by its simplicity—both in data requirements and statistical technique—which allows for its easy calculation from census or census-like microdata across most post-1970 world populations included in IPUMS-I [7,28]. Importantly, PI is based on actual behaviors, not behavioral norms, capturing local/regional family-demographic practices that may enforce or mitigate gender and age hierarchies in access to socially valued resources. The index values (theoretically 0–40) represent absolute measures of gender and age inequalities and, except for the last indicator, do not consider any normative standards or reference categories [138].

#### Reliability.

Most indicators in PI capture gender and generational biases at the household level, significantly impacting women’s access to productive resources and limiting their choices and decision-making abilities (e.g., indicators 1–3 in Fig 2). Other variables serve as proxies for social practices not directly observable in census microdata, such as parental control over marriage (2, 9), female participation in the labor force (4), or gender-discriminatory practices in infancy and childhood at the household level (10–11). Additionally, some indicators capture co-residential configurations that may further constrain women’s freedom (5–7), prospects for educational attainment, or increase their exposure to domestic violence [39,42,81,104,139,140]. Altogether, the indicators and domains of PI align with Walby’s *private/domestic patriarchy* and Kocabıçak’s *modern domestic patriarchy* frameworks, without positing any straightforward causal link to state-level forms of patriarchy, which remain beyond the scope of this paper [38,41,44,48].

**Fig 3:**
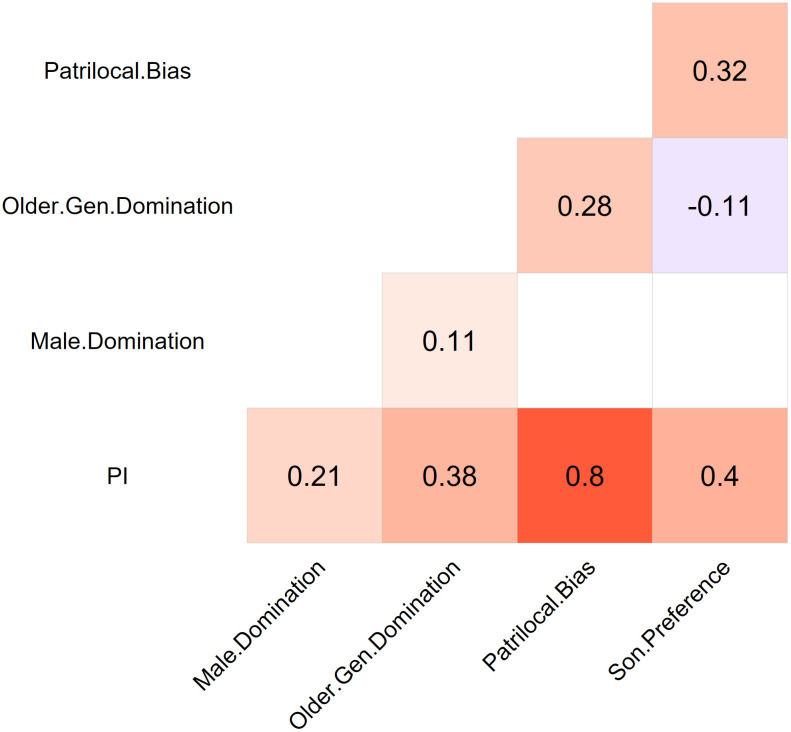
Correlation matrix of the four sub-indices of patriarchy (Kendall’s Tau). Notes: Data source as in Table 1. All colour quadrants indicate significant correlation (p-value<0.05).

A vital axiomatic property of PI is its subgroup decomposability: it can be broken down into its constituent parts (indicators/subindexes) to analyze specific aspects of the construct by examining the individual dimensions that contribute to the overall index, and how these can be interpreted as disempowering factors. This is all the more purposeful given that PI represents a hybrid (reflective-formative) type of index, for which not all its items and dimensions should be required to be highly correlated (see Fig 3) [134,141,142]. Still, PI for Asia exhibits adequate internal consistency reliability, as measured by Guttman’s Lambda-6 (0.75), which was used here because the tau-equivalence condition was not met in the present dataset [143]. This level of reliability is generally considered acceptable and suggests that the items of the index consistently capture the underlying construct of family patriarchy [141,144].

#### Validity.

Internal consistency (see above) is a necessary, though not sufficient, condition for establishing construct validity. Construct validity itself is supported by evidence of convergent validity—the extent to which a measure correlates strongly with other measures of the same or similar constructs—and discriminant (or divergent) validity, which is confirmed when a measure shows low correlations with instruments intended to assess theoretically distinct constructs [145, p.83]. In contexts where directly comparable convergent measures are limited (see above), demonstrating that an instrument both aligns with and diverges from related constructs provides a reasonable framework for establishing overall construct validity [141,145].

**Fig 4:**
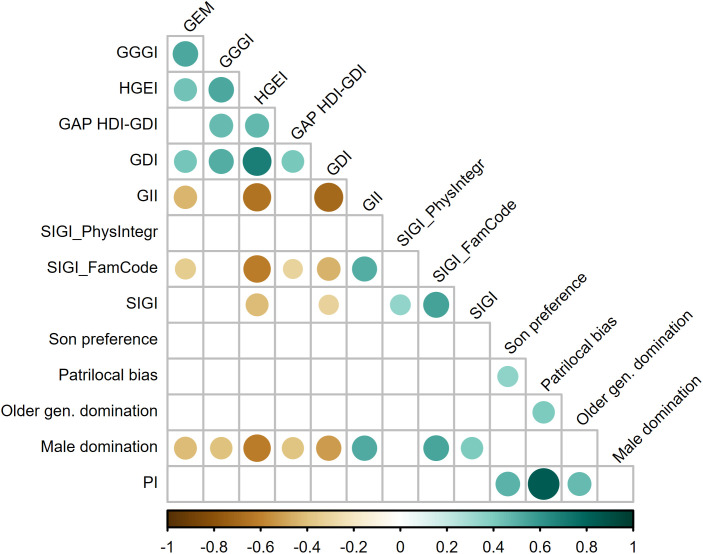
Country-level correlations of PI and its components with main gender (in)equality indices. Notes: Data source for PI and its four domains, as in Table 1. For other indices (in order of appearance): *(GEM) Gender Empowerment Measure* – values are from the closest available date to the census year, i.e., 1995, 2006 (India), or 2009, 2011 (Cambodia; higher value (closer to 1) = greater empowerment of women); see [146–148]. *(GGGI) Global Gender Gap Index* – values are from the closest available date to the census year – i.e., 2006, 2011, 2019, 2020, 2022; higher score (closer to 1.00) = more gender parity) [149]. *(HGEI) Historical Gender Equality Index* – the closest available date (1997–2003) to the census year (higher score = more equality) [150]. *(GDI) Gender Development Index* – exact census years (values below 1 = higher human development for men than women; values above 1 = the opposite) [151]. The gap between HDI and GDI (expressed as a difference) is used following [152], where it is meant to serve as a useful proxy for the developmental costs associated with gender inequality. *(GII) Gender Inequality Index* – exact census years (higher score = lower inequality between women and men) [153]. *(SIGI) Social Institutions and Gender Index* – values are from the closest available date to the census year (i.e., 2009, 2012, 2014; higher score = more gender-based discrimination) [154]*. (SIGI_FamCode) Discriminatory Family Code component of the SIGI* – values are from the closest available date to the census year (i.e., 2009, 2012, 2014; higher score = more discrimination) [154]*. (SIGI_PhysIntegr) Restricted Physical Integrity component of the SIGI* – all values from 2019 (higher score = more restriction) [154]*.*

To evaluate these aspects for the PI, we examined the relationships between the country-level PI (and its main components) and the principal gender (in)equality and empowerment indices available at the country level (e.g., Gender Development Index (GDI), Global Gender Gap Index (GGGI), Gender Inequality Index (GII), Social Institutions and Gender Index (SIGI), Historical Gender Equality Index (HGEI), keeping in mind that none of these indices were designed to capture family patriarchy as such, nor indicators of men’s greater status and control over women at the household level (although few included components partially alike others). Fig 4 using Kendall’s Tau‐b reveals that at the country-level the overall PI does not exhibit significant correlations with established gender inequality indices (whereas one of its subindexes does). This indicates that while certain dimensions of PI align with conventional gender measures, the overall index captures a distinct construct.

However, because these correlations are based on a small sample of 22 countries, it is likely that this aggregation might dilute specific signals that would otherwise be detectable. Unfortunately, our ability to test this hypothesis is significantly constrained by the relative dearth of an appropriate fine-grained but large-scale data. To the best of our knowledge, the only widely available gender (in)equality indicator that corresponds to the maximum number of subnational units for which our PI measure was computed is Subnational Gender Development Index (henceforth, SGDI; see [155]), whereas other subnational data on gender (in)equality in outcomes could be harvested only for a few specific countries. Table 2 provides the results of assessing the relationship of this various measures with PI.

**Table 2:** Correlations of PI with available subnational measures of gender (in)equality.

	Subnational Gender Development Index	Weighted Burden of Patriarchy Index, India 2019−21	Gender Empowerment Measure 2006 (India)	Gender Empowerment Index (Indonesia 2010)	Gender Equality Index, Turkey 2014	Gender Empowerment Index, Turkey 2014
Kendall’s tau_b	−.215^**^	.161	.118	−.030	.355^**^	−.406^**^
Sig. (2-tailed)	.000	.247	.377	.826	.000	.000
**N**	**610**	**28**	**30**	**33**	**81**	**81**

Notes:

Data source for the PI, as in Table 1. A variable *N* indicates challenges in matching all IPUMS-International census regions with other territorial classifications. Wherever possible, indicator values are taken from the census year or the closest available date. Exceptions are the Weighted Burden of Patriarchy (10 years after the focal census year) and Turkey (2014, 14 years after the focal census year), where larger temporal gaps reflect data availability constraints.

Auxiliary secondary data:

*The Subnational Gender Development Index (SGDI)* – a direct ratio of gender-specific Human

Development Indices at the subnational level (higher score = more advantageous position of females relative to males) [156].

*Weighted Burden of Patriarchy Index (India)* – twenty indicators in seven domains (time span 2019–2021): endowments, voice, mobility, son preference, behavioural control, violence, and political participation (higher value = a greater burden of patriarchy, i.e., more intense and widespread gender inequality, across the domains) [157].

*Gender Empowerment Measure (India)* – all values are from 2006 (higher value (closer to 1) = greater empowerment of women) [158]*.*

*Gender Empowerment Index (Indonesia)* – all values are from 2010 (adaptation of the UNDP GEM; higher score = greater empowerment) [159].

*Gender Equality Index (Turkey)* – all values are from 2014 (the earliest available). Consists of 3 sub-indexes and 11 indicators. The 3 sub-indexes, all equally weighted on the index value, are: (i) representation in politics and economy, (ii) participation in production, and (iii) educational attainment (higher score = less equality) [160].

*Gender Empowerment Index (Turkey)* – all values are from 2014; a localized adaptation of the UNDP GII and GEM measures (women’s absolute standing in domains of health, education, labor force participation, political representation; higher score = better representation across domains) [160].

Using the large subnational dataset with 610 value entries, we found a modest negative, but highly significant correlation between PI and SGDI. This negative relationship is theoretically plausible—higher levels of patriarchy measured by PI correspond with lower levels of gender equality in human development as measured by SGDI. In addition, the large number of subnational units provides robust statistical power. The observed association with SGDI provides a hint on convergent validity of PI, suggesting that it sensibly taps into some other but theoretically related gender-(in)equality phenomena.

This observation is supported with two additional local level data for 81 provinces of Turkey. There, the correlations are high and theoretically coherent, despite a certain time stretch from our index’ census date: the Gender Equality Index (GEI) and Gender Empowerment Index (GEM) are strongly inversely correlated—higher GEI values reflect greater inequality, whereas higher GEM scores indicate greater empowerment, and PI exhibits correlations that are in expected directions relative to these indices (more patriarchy indicating more inequality and less empowerment). This reinforces the point that PI does show convergent validity in contexts where the underlying constructs are measured more robustly (or perhaps more similarly) across instruments.

On the other hand, the exploration of the three much smaller subnational datasets (from India and Indonesia) reveals that the correlations between PI and other indices (e.g., Gender Empowerment Measure, Gender Equality Index, etc.) are non-significant. The lack of correlation might suggest that in these contexts the constructs measured by PI diverge from those captured by the other measures. This can be interpreted as an indication that the PI is not merely echoing the same variance as measures of gender (in)equality established for those contexts. However, a notice should be taken to recognize that that these particular analyses have small sample sizes which may affect reliability of the coefficients. And, even if correct, these non-significant correlations might be an asset rather than a liability, suggesting that PI is capturing a distinct facet of patriarchy—especially if other measures of gender (in)equality do not correlate with each other strongly either (e.g., the two Indian measures from Tab 3 are not significantly correlated with each other), indicating that they all tap into different dimensions of gender relations.

Altogether, PI appears to have sound convergent validity in certain contexts (as evidenced by the relationship with SGDI and the Turkish indices) while differentiating itself from other indices in contexts where the constructs measured diverge or where methodological constraints are present. This nuanced pattern is not uncommon in cross-national or subnational comparisons of complex social constructs. Research across a range of disciplines has shown that measurement instruments rarely perform identically across different cultural or regional contexts. These findings underscore that variation in validity is expected when the underlying facets of a complex social construct interact with local historical, social, and cultural forces [161,162].

We should also be cognisant that neither of the significant correlation coefficients was of the size that could suggest redundancy. It is clear that PI is weakly-to-moderately related to some outcome-based gender (in)equality measures, but this correlation is at least far from perfect. This is what we would expect. Clearly, hierarchical gender relationships in domestic groups and family-related demographic behaviour should be an important driver of gender inequality in outcomes; but we would not expect a perfect match [163], and we cannot exclude that PI’s effects may actually be only proximate determinants (covariates) of those outcomes.

## Variability and spatial patterns of patriarchy

### Country-level patterns

**Fig 5:**
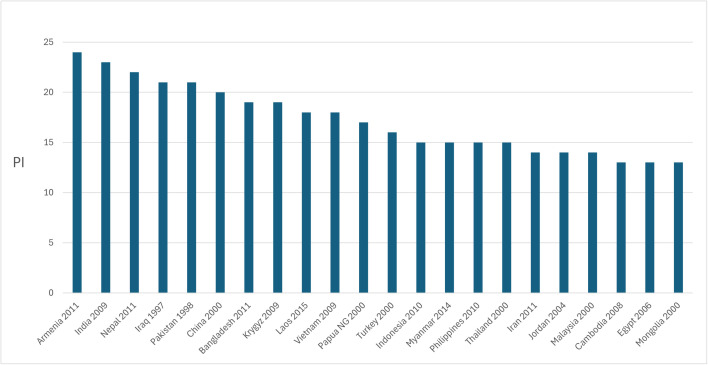
Country-level distribution of PI. Notes: Data source as in Table 1.

PI for 22 Asian countries reveals meaningful variation in the intensity of patriarchal structures at the state level, with index values ranging from 13 to 24 (Fig 5). Armenia (2011) records the highest PI, and Mongolia (2000) the lowest. While the upper end of the distribution includes countries like India, Nepal, Iraq, and Pakistan, and the lower end includes Cambodia and Egypt, nearly half the country-dataset clusters within a narrow band of relatively low values (13–15). This suggests that although variation exists, it is moderate and patterned, rather than evenly or widely dispersed.

The distribution of PI at the state level across the studied region presents few surprises, reinforcing its validity at a broad scale. Countries with high PI scores (such as India, Iraq, and Pakistan) tend to be those with long-standing patriarchal traditions, well-documented in scholarly literature and recognized by institutions monitoring gender inequality [15,90,104]. Similarly, at least some low-PI countries (Cambodia, Malaysia) align with existing understandings of historically entrenched more egalitarian gender regimes in Southeast Asia, often associated with bilateral kinship patterns, the absence of patrilocality, and gender-neutral child preferences [103,164,165]. However, Fig 5 also reveals notable exceptions. Iran, Jordan, and Egypt—despite their apparently established position within the widely referenced *belt of classical patriarchy*—appear unexpectedly among low-PI countries, in some cases ranking even lower than certain Southeast Asian nations (e.g., Laos, which—however, unlike other countries in SEA—is less unambiguously bilateral; see [164–166]).

A full investigation into these anomalies lies beyond the scope of this paper; however, further insights can be gained by reconstructing a more granular analysis of the geographic patterns of PI.

### Subnational patterns

In this section, we present three types of analyses aimed at describing the extent of family patriarchy in our populations at the regional level: 1) descriptive statistics with visual displays, 2) exploratory spatial approaches, and 3) inequality measures.

The overall distribution of PI across 652 regions in the twenty-two countries confirms a moderate spread in the index. Across the entire dataset, the median PI value is 17, and most regions fall within a tight central range between 14 and 19 indexed points. The lower quartile of PI distribution extends to 10, while the upper one reaches near 25. Beyond these bounds, there are several high-end outliers, with some values around 27–28 and one reaching 30, indicating a small subset of regions with particularly high intensity of family patriarchy.

**Fig 6:**
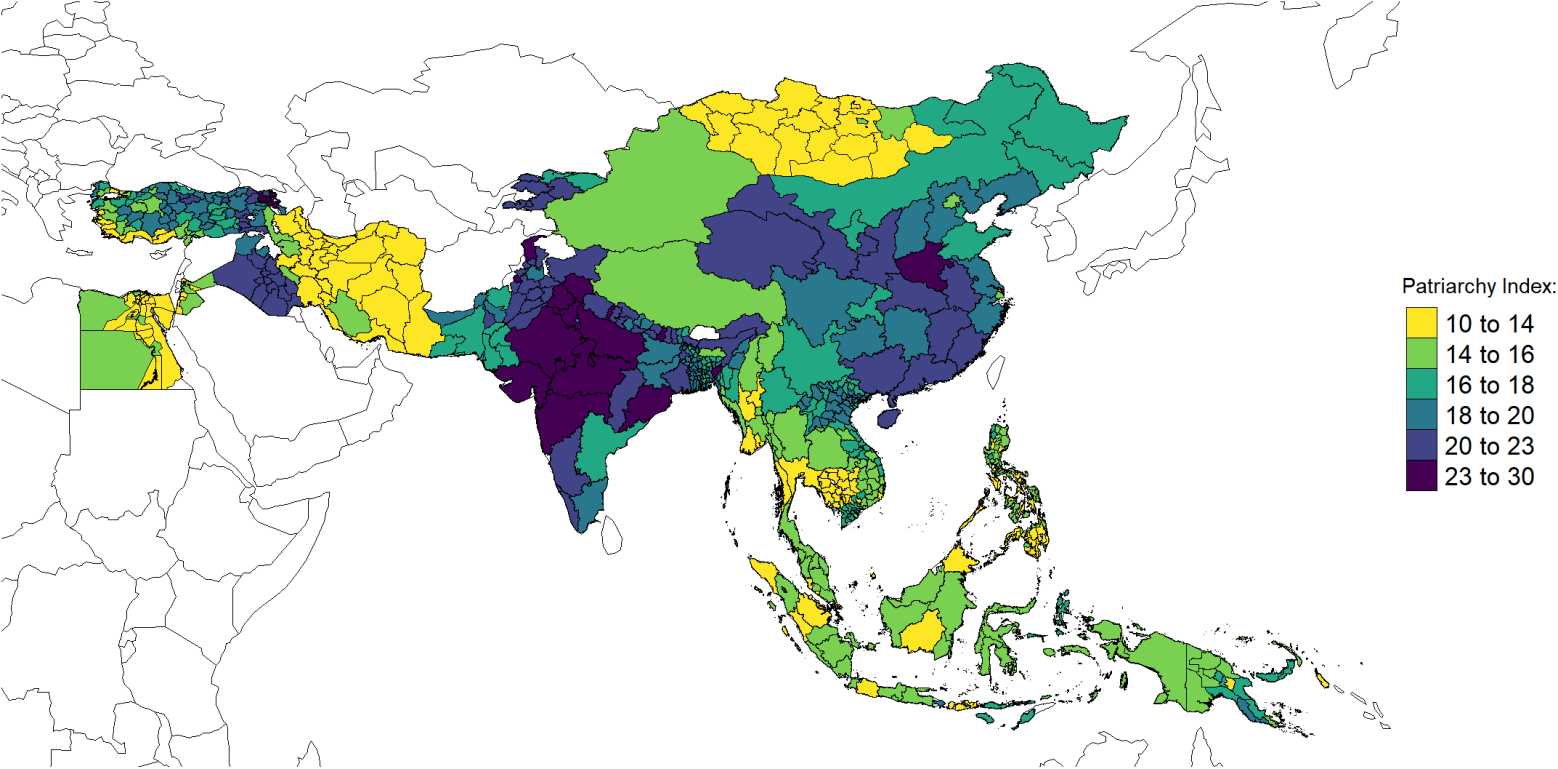
Regional distribution of PI. Notes: Data source and regional divisions as presented in Table 1. Boundaries: Natural Earth, public domain [59].

**Fig 7:**
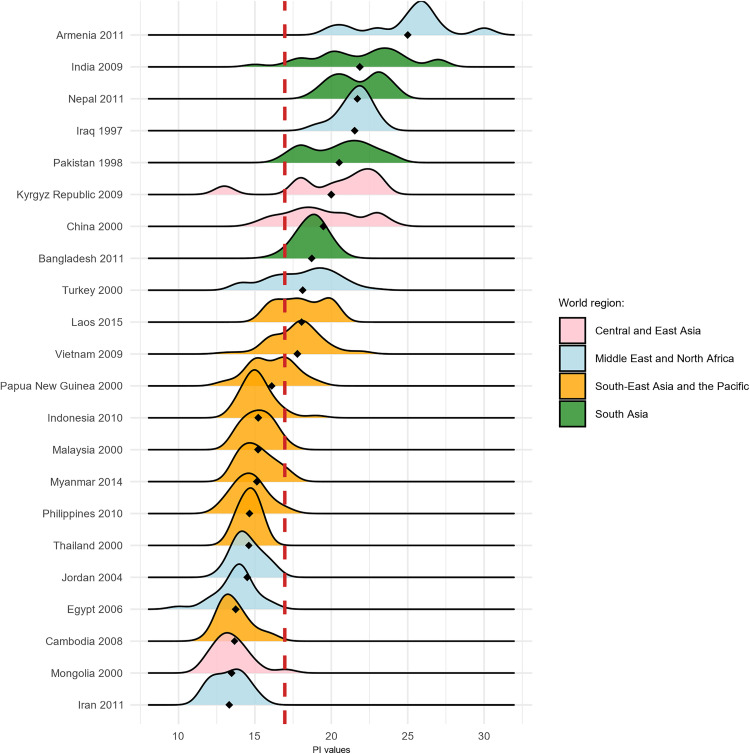
Within-country variation in PI. Notes: Data source and regional divisions as presented in Table 1.

Fig 6 illustrates the spatial distribution of the regional PI values. It is accompanied by Fig 7 which provides a density plot that statistically represents the PI distributions across various countries and regions. Combined, they reveal that Armenia and parts of India are the highest-ranking regions in terms of patriarchal intensity, with maximum scores exceeding 25 out of a total possible range of 40.

More interestingly, these figures also highlight the considerable variability in PI values across the major Asian regions, as well as some of the countries. For example, South Asia (Bangladesh, Pakistan, India and Nepal), while predominantly characterized by high Index values, cannot be regarded as entirely homogeneous. The PI in India, for example, roughly overlays with a well-known north-south division suggested in earlier literature [28,46], likewise as Pakistan [167]. Similarly, the MENA region demonstrates significant heterogeneity, even within in the *classical patriarchal belt*, with Iraq and Pakistan exhibiting the highest PI values in the region, while others falling considerably below the red dashed reference line indicating the median PI value. In this regard, the confrontation of Iran of 2011 with Iraq a dozen or so years earlier is particularly striking. Whereas the former recorded one of the lowest average PI within the entire dataset—in fact, an unexpected finding, considering its theocratic regime, gender-segregated institutions, and strict dress codes prevailing int the country, neighbouring Iraq exhibits some of the highest PI values. The divergence between Iran and Iraq may partly reflect temporal variation, as the Iraqi data stem from 1997 and the Iranian from 2011, thus capturing distinct demographic stages and political cum institutional contexts. Iran’s rapid fertility decline and family change by the 2000s contrasts with Iraq’s earlier, more patriarchal context, shaped at the time by UN sanctions, conflict, and international isolation that constrained the modernization of social institutions and women’s education [18,50,168].

A notable contrast also emerges within the Central and East Asian countries—namely Kyrgyzstan, Mongolia, and China. While the region is generally characterized by high PI values, Mongolia stands out from its neighbours due to both its significantly lower patriarchy scores and its remarkable uniformity. A recent analysis by Torabi [169] identifies Mongolia as one of the Asian countries with the lowest reported rates of early marriage, which may contribute to its comparatively low PI values. Additionally, several scholars have suggested that herding societies tend to be more individualistic—and potentially less patriarchal—than agricultural societies [170]. China, though generally leaning toward relatively higher index values, exhibits a dramatic divergence between the core Han Chinese regions, where PI reaches its highest levels, and the Autonomous Regions, where it is considerably lower. Notably, higher PI values appear to correspond closely with regions that exhibit a long-standing imbalance in the sex ratio at birth [171; 132]. In contrast, the South-East Asian and Pacific regions display greater homogeneity in index scores, with Cambodia serving as a prime example. Similarly, island-based or insular areas within the Asia-Pacific region also show relatively lower and more concentrated PI distributions.

Neither the map nor the density plot reveals a clear east–west or north–south gradient. Lower PI values (represented as yellow and light green patches on the map) appear in geographically dispersed areas located mostly in peripheral positions on the map. These include areas in the western part of the mapped region (e.g., Turkey and Armenia), the center (e.g., parts of China), and various sites in the southeast. These observations challenge existing approaches to intra-Asian gender inequality, revealing that levels of patriarchy in the *classical patriarchal belt* are neither exceptionally high nor indicative of a monolithic patriarchal block [15].

However, as a purely visual inspection of distribution maps can be unreliable, the next step is to use the exploratory spatial data analysis (ESDA) tools to formally assess the global spatial patterns of the PI distribution in our data. This approach reveals an extremely strong clustering tendency in the spatial spread of patriarchy with a Moran’s *I* of PI = 0.768 (p < 0.001; a spatial lag using seven nearest neighbors was used). This value indicates that neighbouring regions in our dataset tend to have similar values – i.e., areas with high PI values are clustered near other areas with high values, and areas with low PI values are clustered near other areas with low values.

**Fig 8:**
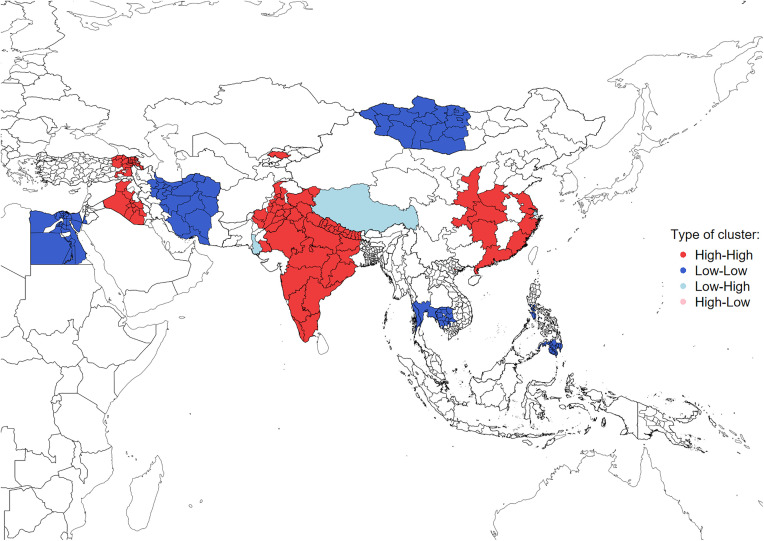
LISA significant (p < 0.001) clusters for the distribution of the Asian PI. Notes: Data source and regional divisions as presented in Table 1. Boundaries: Natural Earth, public domain [59].

Identification of spatial clusters and outliers can be formalized by the analysis of Local Indicator of Spatial Autocorrelation (LISA) (Fig 8). The null hypothesis underlying the calculation is that the values of interest exhibit a random spatial pattern – i.e., that the spatial variation in patriarchy patterns does not deviate from the spatial variation we would expect to observe by chance. Accordingly, the LISA clusters are recognised if their values are significant at least at the 95% level, indicating the regions that have the greatest influence on the global autocorrelation result [172].

Fig 8 confirms that at least part of the groupings observed in distribution of PI is non-random. The significant clusters of high patriarchy encompass most of Indian Subcontinent, easternmost regions of Turkey together with Armenia and Iraq, Kyrgyzstan, as well as south-eastern China. On the other hand, the low patriarchy clusters are identified in Egypt, Iran and most of Mongolia. The Moran scatterplot (not presented) shows that there are only few cases of spatial outliers (the observations atypical given the values of PI in the surrounding neighbors), which are: Xizang Autonomous Region (Tibet) and Shanghai in China, Hyderabad in Pakistan, and Bishkek in Kyrgyzstan.

Finally, in order to enrich our understanding of the amount of variation in different segments of our data (countries, geographical/administrative regions) we computed the *Theil*-index of inequality and its decomposition [173]. This analysis uncovers a key feature of the PI distribution across both time and space: familial patriarchy values are predominantly clustered within countries, rather than within lower-level groupings (both data polygons in macroregions and countries in macroregions show higher within than between inequality) (Table 3). The polarization index of 2.98 describing the configuration where data points (regions) are nested in countries (as defined by the date of the source used) shows that the bulk of inequality at this granularity comes from differences between the countries rather than within them (75% of the variation in the data occurs between the countries). Given the significant geographical and cultural variability of the focal area, this may strengthen hypotheses on the role of country-related factors (e.g., policy, institutions, historical endowment, social arrangements) in shaping the patriarchal outlook of Asia [174].

**Table 3:** Theil’s inequality measures in PI distribution across Asia.

Parameter	Data points in countries	Data points in geographical regions	Countries in geographical regions
Theil Index	0.017	0.017	0.017
Between Component	0.013	0.005	0.007
Within Component	0.004	0.012	0.010
Polarization Index	2.973	0.409	0.696
% of between inequality	74.83	29.02	41.03%
N in n	652 in 22	652 in 4	22 in 4

Notes:

Data source as in Table 1. Regional divisions as in Table 1 and Fig 1.

## Instrumental value of PI

Previous analyses demonstrated that higher levels of domestic patriarchy generally correspond to less favorable gender outcomes at the regional level. Building on this, we provide a targeted assessment of the PI’s instrumental value —that is, its ability to explain or predict variation in broader gender outcomes beyond the family sphere. If PI reliably captures domestic patriarchy, it may—despite necessary caveats—offer insights into women’s positioning across other/broader societal domains [39, p.358].

Following [65], we provisionally test the PI’s instrumental value by analyzing cross-regional variations in four different outcome measures of gender inequalities. To merely signal lines of inquiry that may be pursued in more detailed analyses, we begin by focusing on three of the most salient cross-national indicators of women’s status: female (relative) labor force participation (FLFP), gender gaps in life expectancy (GGLE; see [175]), and the female-to-male ratio in expected years of schooling (EYS). To this, we add a measure of gender disparities in basic numeracy skills, operationalized through age-heaping (i.e., the tendency to concentrate census reported ages at preferred digits such as 0 or 5, but here extended to capture clustering at all digits) [176]. Finally, we consider the Cross-Gender Friending Ratio (CGFR), recently introduced by Bailey et al. (2025), which captures the extent of Facebook-based social connectivity across gender lines. The CGFR is derived from a snapshot of more than 1.38 trillion social ties among 1.8 billion users aged 18–65 who were active on the platform in the 30 days prior to January 9, 2025, spanning nearly 200 countries and their territories [177].

In each case, we examine the degree to which these disparities are explained by variation in PI or its constituent components, while controlling for Gross National Income (GNI) per capita and the share of the population residing in urban areas, in order to isolate the independent effect of patriarchal structures. All model coefficients are derived from a stepwise decomposition of a composite index using OLS regression. The analysis first models the effect of the overall PI on the outcome variable, followed by separate regressions replacing PI with its individual subcomponents, while consistently including the same control variables across all models.

**Fig 9:**
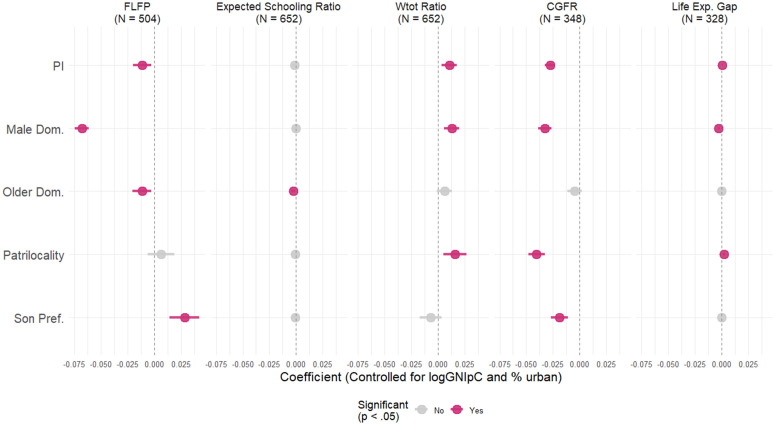
Estimated effects of PI and its subcomponents on selected gender (in)equality outcomes (OLS). Notes: The variation in the number of observations across models stems from differences in data availability. In some cases, certain regions or entire countries were excluded due to missing or non-harmonized data, or because available data referred to time periods too distant from the reference year. Additionally, some variables were only reported at broader territorial levels than others, limiting consistent coverage across all units. For full model results, see S1 Tables. Response variables: *FLFP* – relative female labor force participation (absolute_female/ absolute_men); exact census years as PI. *Expected Schooling Ratio* – Female/Male ratio in expected years of schooling (EYS), where EYS is defined as the number of years of schooling a child of school entrance age can expect to receive, if prevailing patterns of age-specific enrolment rates persist throughout the child’s schooling life. Values are from the closest available date to the census. *W*_*tot*_ *ratio* – the Female/Male ratio in age-heaping as measured by the Total Modified Whipple’s Index (W_*tot*_) [178]. *CGFR* – cross gender friendship ratio; the share of female friends among male users on Facebook divided by the share of female friends among female users in a given location. The closer the value of the ratio to 1 the more men and women form equal shares of their FB ties with women. The data used here reflects friendships as of January 30, 2025. The available data cover 348 subnational regions from our collection, with approximately 75% drawn from Philippines, Turkey, Vietnam, India, and Indonesia. Countries where Facebook is inaccessible (e.g., China, Iran) are not included [177]. *Life expectancy at birth(e*_*0*_*) gap* – the relative gender gap in e_0_ with respect to men’s e_0_ (Women’s e_0_– Men’s e_0_)/Men’s e_0_) [175]. Values are from the closest available date to the census. Control variables: *% urban* – proportion of the households in a region *considered* as located in a place labelled as urban or rural. *GNI* – Log Gross National Income per capita in thousands of US Dollars (2011 PPP). Sources: for FLFP, W_*tot*_ and % urban – computed, respectively, from IPUMS-I variables LABFORCE, URBAN, and AGE for the same censuses as the PI; for EYS ratio, e_0_ gap, see [179]. % urban for Mongolia 2000, see [180].

The main findings from Fig 9 demonstrate that PI is significantly associated with nearly all external measures of gender inequality, even after controlling for key structural factors. These associations generally follow the expected pattern—higher levels of patriarchy correlate with greater gender disparities—offering encouraging validation of the PI’s empirical utility. While all four sub-indices show meaningful correlations with some gender inequality outcomes, the strength and direction of these associations vary. For instance, relative female labor force participation declines most sharply in relation to Male Dominance, yet unexpectedly rises with Son Preference, suggesting multidimensional effects within patriarchal systems. The F/M age-heaping ratio and CGFR display the most robust and directionally aligned associations across both the PI and its subcomponents, suggesting that higher levels of patriarchy are associated with reduced relative female numeracy and greater gender-based segregation in Facebook network connections. While both outcomes are amenable to interpretation as conditioned by embedded patriarchal norms, the Facebook–PI correlation warrants more cautious interpretation. The Facebook CGFR data are considerably more recent than the census years underlying the PI (with no pan-Asian PI values available for 2025 in IPUMS-I), and the correlation is based on only 348 subnational regions, approximately 75% of which are drawn from five countries—Philippines, Turkey, Vietnam, India, and Indonesia. Although not globally representative, this subsample nonetheless reflects meaningful contextual diversity, encompassing countries traditionally associated with both high and low levels of gender inequality (see Sample characteristics section). Precisely because of these contrasts, we regard attention to the PI–Facebook correlation as analytically salient, underscoring its heuristic value in linking offline social structures with online behaviour.

Conversely, associations with gender gaps in life expectancy are less consistent: while the overall PI and Patrilocality show small positive effects, Male Dominance is moderately and negatively associated with the gap—potentially reflecting an erosion of women’s longevity advantage in highly patriarchal contexts. These results support the view that no single measure can fully capture the complexity of gender regimes. Instead, distinct domains of family-based patriarchy exert varying levels—and possibly opposing directions—of influence on different aspects of gender inequality, underscoring the need for disaggregated analysis in both research and policy.

However preliminary, these findings suggest that, beyond its role as a diagnostic tool for gender inequities in domestic and family dynamics, PI can serve as an important correlate—and potentially a predictor—of broader gender (in)equalities extending beyond the domestic sphere. While this aspect requires further in-depth research that accounts for diverse social, economic, and cultural contexts, this dual function of PI may well signal its analytical value, particularly in its capacity to bridge private and public realms in gender analysis.

## Discussion and conclusion

This study introduces the Patriarchy Index, a novel composite measure for assessing gendered power asymmetries embedded in family systems. Drawing on harmonized census microdata from multiple Asian countries, the index addresses a persistent gap in global gender metrics by capturing household-level dynamics that shape female autonomy—particularly where co-residence norms structure authority across generations.

Unlike conventional indicators that emphasize well-being outcomes or formal legal constraints, the PI proxies deeply rooted informal institutions using observable demographic configurations. Its methodological economy—relying on standardized variables such as age at marriage, household composition, and kinship ties—makes it cost-effective and scalable, especially in low- and middle-income countries (LMICs). Notably, to date only the Gender Development Index (GDI) offers similarly disaggregated gender assessments, though it does not attend to domestic organization or kin-based power hierarchies. The PI has already motivated analogous efforts in Africa, India, and Italy, highlighting its relevance and portability [28,131,181].

Applying PI across multiple Asian contexts reveals significant spatial variation in patriarchal intensity. Some regions display markedly high scores suggestive of entrenched family-based control, while others exhibit greater internal diversity, indicating localized shifts in household power relations. Importantly, the index’s empirical relevance is supported through a three-tiered validation strategy: *convergent validity* is demonstrated through its alignment with established measures such as the GDI; *discriminant validity* is shown through its capacity to identify subnational gender disparities that conventional indicators overlook; and *instrumental relevance* is evidenced by its statistically significant association with several external measures of gender inequality, even after adjusting for main structural variables.

By moving beyond the dyadic husband–wife unit and incorporating more extended kinship networks, PI offers a more nuanced lens to understand how household arrangements condition female agency. This conceptual realignment is especially relevant in Asia, where patrilocality and generational hierarchies often exert stronger constraints than spousal inequality alone. In doing so, the PI complements rather than replaces existing indices, and serves as a practical diagnostic tool for researchers and policymakers alike.

While traditional gender indicators often capture public-facing achievements in education or employment, the PI highlights subtler, yet persistent, forms of private-sphere inequality. In many parts of Asia, patriarchal control remains organized around extended kinship and residential arrangements that are rarely accounted for in global indices. This gap is particularly consequential in countries where high female participation in politics or the public sector coexists with diminished autonomy in the domestic sphere [84–86]. As numerous studies have shown [39,75–77,81], interventions aimed at advancing gender equality without attending to family-level power structures risk misdiagnosing both the origins and persistence of inequality. By centering household demography, PI helps reconcile the epistemic disjuncture between public empowerment and private subordination.

The PI’s ability to generate national and subnational estimates also opens tangible policy pathways. In regions characterized by high PI scores, policy attention might usefully focus on intrafamily legal protections, targeted cash transfers to female-headed households, or the expansion of shelter and mobility infrastructure for women isolated by household control. In locales with internal polarization—where patriarchal norms vary widely between communities—interventions could include localized behavioral campaigns, social norm transformation efforts, or decentralization of women’s legal aid services. These applications are particularly relevant for LMICs where administrative and statistical capacity may limit more resource-intensive survey-based approaches. As such, PI offers a scalable diagnostic for both macro-level targeting and community-sensitive intervention design.

Several limitations merit acknowledgment. First, the construction of PI depends on the availability and comparability of census indicators, which vary across countries and census years [182]. Several countries had to be excluded from the present analysis because key variables were missing or inconsistently coded, raising the possibility of measurement error; even for the data ultimately included, adaptations were necessary. This underscores the uneven quality and coverage of census microdata, which remains a structural challenge for comparative research of this kind. Future work should audit existing collections for the construction of specific variables before extending the PI framework to additional geographical contexts.

In particular, data from the Demographic and Health Surveys (DHS) present a potentially valuable avenue for extending the PI framework to regions not covered by standard IPUMS census microdata. However, while DHS surveys provide rich, cross-nationally comparable information on marriage, fertility, household headship, and child sex composition, they lack the kinship detail required to reproduce several of the original PI indicators. Preliminary screening suggests that at least four of the eleven PI indicators cannot be computed with DHS data because of the surveys’ limited kinship detail (no/limited information on in-law ties or inter-household links; no reliable distinction between kin and non-kin; omission of lateral relatives such as siblings or cousins; exclusion of non-kin roles such as lodgers or servants). These constraints imply that any DHS-based adaptation of the PI would require the careful development of proxies or modified indicators to preserve conceptual fidelity.

Second, while the PI captures domestic constraints at the household level, it does not encompass all domains of women’s autonomy, such as community participation, political representation, or legal rights. Recent extensions of the PI illustrate promising directions in this regard. For example, [28] introduced additional components for India that capture measures of *socio-economic domination*, such as the relative education of wives and women’s participation in professional occupations. By contrast, [181] expanded the index for Italy with indicators reflecting *labour market participation* (including gender gaps in involuntary part-time work), *political representation* (proportion of women elected), and *cultural or attitudinal dimensions* (beliefs about abortion, prevalence of abuse). These innovations demonstrate the open-ended structure of the PI, which can be adapted to capture nationally specific manifestations of patriarchy. At the same time, they highlight a methodological trade-off: while such extensions enrich country-level analyses, their incorporation into large-scale comparative frameworks raises questions of feasibility, data availability, and the time economy of research design. Moreover, the very openness of the PI composition requires caution. If too many additional aspects are incorporated—especially those endogenous to family patriarchy itself, the otherwise fruitful distinction between family (private) and public patriarchy [38,72] risks being blurred, with the consequence that the analytical clarity of the measure is undermined.

Third, the broader instrumental relevance of the PI remains to be tested. In the present study, statistically significant associations with several external measures of gender inequality (e.g., relative female labour force participation) remained robust even after adjusting for structural conditions, but the model was relative simple. The recent study by Singh et al. [28] reinforces this line of inquiry by demonstrating how patriarchal family systems can be operationalized to explain demographic outcomes beyond fertility alone, linking kinship structures to survival disadvantages for girls in India. By explicitly connecting household-level patriarchal practices with measurable demographic consequences, their work shows that patriarchal intensity is not merely a cultural descriptor but a structural determinant with predictive power across multiple demographic domains. A similar PI framework could be adapted for analytic strategies in other areas where subnational variation is critical—such as migration, gender preferences for children, family dynamics, and health behavior—as well as at larger, cross-national scales. Taken together, these strands of evidence point toward a broader research agenda in which the PI functions both as a diagnostic tool for subnational disparities and as a theoretically grounded instrument for explaining the demographic consequences of patriarchal family systems [163].

Finally, the cross-sectional research design applied in this paper does not permit the analysis of temporal dynamics. Although earlier census rounds could, in principle, be used to trace longitudinal trends, our exploration of IPUMS data reveals that, for a substantial number of countries, earlier enumerations lack the necessary information to compute the index. As a result, the present analysis provides a valuable snapshot of patriarchal intensity but cannot fully capture its evolution over time. This limitation may be addressed in future country-specific studies, particularly in contexts where harmonized census microdata are available across multiple decades (for an illustration of such longitudinal potential, see IPUMS US data). At the same time, any longitudinal extension of the framework must remain sensitive to changes in census definitions of the household [96,182], a well-documented source of comparability problems in international census harmonization.

## Supporting information

S1 Text Adjustments to the Patriarchy Index for IPUMS-International data. (DOCX)

S1 Tables Full regression results for Fig 9. (DOCX)

S1 Data Asian Patriarchy Data. (XLSX)
