# Supplementary material for: The Patriarchy Index for Asia: A new tool for subnational analysis of gender inequalities
Source: PLoS One. 2026 Jan 6;21(1):e0339587. doi: 10.1371/journal.pone.0339587 (PMC12774345; doi:10.1371/journal.pone.0339587)
Supplement: S1 Text — (DOCX) [file pone.0339587.s001.docx]

**S1 Text. Adjustments to the Patriarchy Index for IPUMS-International data**

For the purposes of this paper, the original computational rules for the PI (Gruber and Szołtysek, 2016) had to be adjusted due to some information structure differences in IPUMS-International files. This is because the IPUMS International data do not provide an elaborated number of kinship categories for all files, and if there are elaborated kinship categories, they are not consistent for all available files (variable RELATED). Only a reduced number is available for all files (variable RELATE): head; spouse/partner; child; other relative; non-relative; other relative or non-relative; unknown.

The presence of the pointer variables (MOMLOC, POPLOC, SPLOC) enables the reconstruction of a large part of the lineal kinship relationships (ancestors and descendants). The main disadvantage is that it is not possible to bridge a missing generation, e.g. from ego to grandchild, if the parents of the grandchild are missing.

With the pointers to the parents, we can reconstruct the parents and grandparents of Ego and his spouse. Using the pointers to these parents, we can also reconstruct the other children of these parents: Uncles and aunts, siblings and children. The following generations can also be found: Grandchildren, nephews/nieces and cousins or great-grandchildren, great-nephews and great-cousins. The references to the spouses make it possible to reconstruct the spouses of the reconstructed relatives. These reconstructions were verified using a subsample of the Mosaic data files (N = 446,983 individuals) that already had these pointer variables by comparing the reconstructed relationships with the coded relationships. Overall, this provides confidence that the reconstructed links correctly reflect the majority of potentially existing relationships (calculations available on request). See **Table A**.

**Table A. The components and characteristics of the abridged version of the Patriarchy Index**

| **Domain** | **Components** | **Original definition** | **Relationship with patriarchy** | **Adaptation needed** |
| --- | --- | --- | --- | --- |
| **MALE DOMINATION** | Female household heads | The proportion of all female household heads (20+ years) among all adult heads of family households | Negative | No |
|  | Young brides | The proportion of ever-married women in the age group 15-19 years | Positive | No |
|  | Older wives | The proportion of all wives who are older than their husbands among all couples for whom the ages of both spouses are known | Negative | The SPLOC-variable is used for identifying spouses |
|  | Female non-kin | The proportion of women aged 20-34 years who live as non-kin, usually as lodgers or servants | Negative | No |
| **GENERATIONAL DOMINATION** | Younger household head | The proportion of men aged 65+ years living in a household headed by a male household head of a younger generation | Negative | A younger age is used instead of a younger generation |
|  | Neolocal | The proportion of male household heads living without any relatives except spouses/children among ever-married men aged 20-29 years | Negative | No |
|  | Lateral | The proportion of people aged 65+ years living with at least one lateral relative in the household | Positive | All relatives, who are not identified as lineal relatives are treated as lateral relatives. Identification of kinship relationships is based on pointer variables (POPLOC, MOMLOC, SPLOC) |
|  | Elderly without kin | The proportion of people aged 65+ years living without relatives or spouses | Negative | Identification of kinship relationships is based on pointer variables (POPLOC, MOMLOC, SPLOC) |
| **PATRILOCALITY** | Married daughters living patrilocally | The proportion of ever-married women aged 15 to 30 years who reside with at least one adult male relative of their husband or his mother | Positive | The husband lives with at least one parent and the wife does not live with any parent |
| **SON PREFERENCE** | Boy as last child | The proportion of boys among the last children (if the last child is one of a set of siblings of both sexes, he or she will be excluded from the analysis). | Positive | The analysis is based on the POPLOC variable and therefore groups of siblings. It is no longer restricted to households heads. It is restricted to POPLOC, because the inclusion of MOMLOC, too, would duplicate the children with both parents in the census while those children with only one parent in the census would be only once in the analysis |
|  | Sex ratio | The sex ration (boys to 100 girls) in the youngest age-group (0-4 years old) | Positive | No |
