# Supplementary material for: The Patriarchy Index for Asia: A new tool for subnational analysis of gender inequalities
Source: PLoS One. 2026 Jan 6;21(1):e0339587. doi: 10.1371/journal.pone.0339587 (PMC12774345; doi:10.1371/journal.pone.0339587)
Supplement: S1 Tables — (DOCX) [file pone.0339587.s002.docx]

**S2 Tables. Full regression results for Figure 9.**

|  | **relative female LFP** | | **relative female LFP** | | **relative female LFP** | | **relative female LFP** | | **relative female LFP** | |
| --- | --- | --- | --- | --- | --- | --- | --- | --- | --- | --- |
| *Predictors* | *Estimates* | *p* | *Estimates* | *p* | *Estimates* | *p* | *Estimates* | *p* | *Estimates* | *p* |
| (Intercept) | 0.6595 | **0.021** | 2.2629 | **<0.001** | 1.0077 | **0.003** | 0.4927 | 0.077 | 0.6382 | **0.021** |
| GNI | 0.0265 | 0.446 | 0.0300 | 0.237 | -0.0052 | 0.887 | 0.0187 | 0.594 | -0.0055 | 0.876 |
| % urban | -0.0037 | **<0.001** | -0.0047 | **<0.001** | -0.0032 | **0.001** | -0.0032 | **0.001** | -0.0025 | **0.008** |
| PI | -0.0112 | **0.010** |  |  |  |  |  |  |  |  |
| Male Dom. |  |  | -0.0680 | **<0.001** |  |  |  |  |  |  |
| Older Dom. |  |  |  |  | -0.0115 | **0.011** |  |  |  |  |
| Patrilocality |  |  |  |  |  |  | 0.0062 | 0.328 |  |  |
| Son Pref. |  |  |  |  |  |  |  |  | 0.0284 | **<0.001** |
| Observations | 504 | | 504 | | 504 | | 504 | | 504 | |
| R^2^ / R^2^ adjusted | 0.061 / 0.056 | | 0.499 / 0.496 | | 0.061 / 0.055 | | 0.050 / 0.045 | | 0.078 / 0.073 | |

|  | **Wtot ratio** | | **Wtot ratio** | | **Wtot ratio** | | **Wtot ratio** | | **Wtot ratio** | |
| --- | --- | --- | --- | --- | --- | --- | --- | --- | --- | --- |
| *Predictors* | *Estimates* | *p* | *Estimates* | *p* | *Estimates* | *p* | *Estimates* | *p* | *Estimates* | *p* |
| (Intercept) | 0.5578 | **0.019** | 0.4297 | 0.077 | 0.5045 | 0.067 | 0.7348 | **0.001** | 0.7766 | **0.001** |
| GNI | 0.0357 | 0.208 | 0.0327 | 0.247 | 0.0466 | 0.115 | 0.0309 | 0.276 | 0.0353 | 0.217 |
| % urban | 0.0010 | 0.195 | 0.0010 | 0.190 | 0.0006 | 0.408 | 0.0009 | 0.217 | 0.0006 | 0.457 |
| PI | 0.0108 | **0.003** |  |  |  |  |  |  |  |  |
| Male Dom. |  |  | 0.0130 | **<0.001** |  |  |  |  |  |  |
| Older Dom. |  |  |  |  | 0.0063 | 0.086 |  |  |  |  |
| Patrilocality |  |  |  |  |  |  | 0.0161 | **0.003** |  |  |
| Son Pref. |  |  |  |  |  |  |  |  | -0.0066 | 0.212 |
| Observations | 652 | | 652 | | 652 | | 652 | | 652 | |
| R^2^ / R^2^ adjusted | 0.027 / 0.023 | | 0.034 / 0.030 | | 0.018 / 0.014 | | 0.027 / 0.022 | | 0.016 / 0.012 | |

|  | **school ratio nearest year** | | **school ratio nearest year** | | | **school ratio nearest year** | | **school ratio nearest year** | | **school ratio nearest year** | |
| --- | --- | --- | --- | --- | --- | --- | --- | --- | --- | --- | --- |
| *Predictors* | *Estimates* | *p* | *Estimates* | | *p* | *Estimates* | *p* | *Estimates* | *p* | *Estimates* | *p* |
| (Intercept) | 1.0617 | **<0.001** | 1.0473 | | **<0.001** | 1.1459 | **<0.001** | 1.0389 | **<0.001** | 1.0367 | **<0.001** |
| GNI | -0.0050 | 0.544 | -0.0047 | | 0.569 | -0.0102 | 0.237 | -0.0046 | 0.580 | -0.0045 | 0.587 |
| % urban | -0.0005 | **0.015** | -0.0005 | | **0.020** | -0.0005 | **0.030** | -0.0005 | **0.019** | -0.0005 | **0.019** |
| PI | -0.0013 | 0.237 |  | |  |  |  |  |  |  |  |
| Male Dom. |  |  | -0.0004 | | 0.694 |  |  |  |  |  |  |
| Older Dom. |  |  |  | |  | -0.0025 | **0.017** |  |  |  |  |
| Patrilocality |  |  |  | |  |  |  | -0.0010 | 0.535 |  |  |
| Son Pref. |  |  |  | |  |  |  |  |  | -0.0008 | 0.599 |
| Observations | 652 | | 652 | | | 652 | | 652 | | 652 | |
| R^2^ / R^2^ adjusted | 0.029 / 0.025 | | 0.028 / 0.023 | | | 0.036 / 0.031 | | 0.028 / 0.023 | | 0.028 / 0.023 | |
|  | **cgfr 100** | | **cgfr 100** | | **cgfr 100** | | **cgfr 100** | | **cgfr 100** | |  |
| *Predictors* | *Estimates* | *p* | *Estimates* | *p* | *Estimates* | *p* | *Estimates* | *p* | *Estimates* | *p* |  |
| (Intercept) | 2.7829 | **<0.001** | 3.4298 | **<0.001** | 2.8511 | **<0.001** | 2.3179 | **<0.001** | 2.5364 | **<0.001** |  |
| GNI | -0.1997 | **<0.001** | -0.2392 | **<0.001** | -0.2533 | **<0.001** | -0.1851 | **<0.001** | -0.2225 | **<0.001** |  |
| % urban | -0.0020 | **<0.001** | -0.0009 | 0.093 | -0.0007 | 0.251 | -0.0021 | **<0.001** | -0.0016 | **0.015** |  |
| PI | -0.0276 | **<0.001** |  |  |  |  |  |  |  |  |  |
| Male Dom. |  |  | -0.0323 | **<0.001** |  |  |  |  |  |  |  |
| Older Dom. |  |  |  |  | -0.0048 | 0.167 |  |  |  |  |  |
| Patrilocality |  |  |  |  |  |  | -0.0404 | **<0.001** |  |  |  |
| Son Pref. |  |  |  |  |  |  |  |  | -0.0187 | **<0.001** |  |
| Observations | 348 | | 348 | | 348 | | 348 | | 348 | |  |
| R^2^ / R^2^ adjusted | 0.581 / 0.577 | | 0.561 / 0.557 | | 0.432 / 0.427 | | 0.567 / 0.563 | | 0.463 / 0.458 | |  |

|  | **life expectancy gap nearest year mean** | | **life expectancy gap nearest year mean** | | **life expectancy gap nearest year mean** | | **life expectancy gap nearest year mean** | | **life expectancy gap nearest year mean** | |
| --- | --- | --- | --- | --- | --- | --- | --- | --- | --- | --- |
| *Predictors* | *Estimates* | *p* | *Estimates* | *p* | *Estimates* | *p* | *Estimates* | *p* | *Estimates* | *p* |
| (Intercept) | -0.0280 | 0.292 | 0.0402 | 0.085 | -0.0164 | 0.556 | -0.0362 | 0.135 | -0.0045 | 0.852 |
| GNI | 0.0092 | **0.003** | 0.0105 | **<0.001** | 0.0084 | **0.006** | 0.0110 | **<0.001** | 0.0080 | **0.008** |
| % urban | 0.0000 | 0.564 | -0.0000 | 0.699 | 0.0000 | 0.573 | 0.0000 | 0.790 | 0.0000 | 0.531 |
| PI | 0.0008 | **0.037** |  |  |  |  |  |  |  |  |
| Male Dom. |  |  | -0.0024 | **<0.001** |  |  |  |  |  |  |
| Older Dom. |  |  |  |  | 0.0004 | 0.314 |  |  |  |  |
| Patrilocality |  |  |  |  |  |  | 0.0024 | **<0.001** |  |  |
| Son Pref. |  |  |  |  |  |  |  |  | 0.0003 | 0.517 |
| Observations | 328 | | 328 | | 328 | | 328 | | 328 | |
| R^2^ / R^2^ adjusted | 0.086 / 0.078 | | 0.172 / 0.164 | | 0.077 / 0.068 | | 0.128 / 0.119 | | 0.075 / 0.066 | |
